# Supporting physical activity for mobility in older adults with mobility limitations (SuPA Mobility): study protocol for a randomized controlled trial

**DOI:** 10.1186/s13063-023-07798-9

**Published:** 2023-11-28

**Authors:** Jordyn Rice, Linda C. Li, Jennifer C. Davis, Marco Pahor, Kenneth Madden, Nathan Wei, Hubert Wong, Dawn A. Skelton, Sioban McCormick, Ryan S. Falck, Cindy K. Barha, Ryan E. Rhodes, Sohail Loomba, Mohsen Sadatsafavi, Teresa Liu-Ambrose

**Affiliations:** 1 University of British Columbia, 2329 West Mall, Vancouver, BC V6T 1Z4 Canada; 2 University of British Columbia-Okanagan, 3333 University Way, Kelowna, BC V1V 1V7 Canada; 3 University of Florida, Gainesville, FL 32611 USA; 4 Glasgow Caledonian University, Cowcaddens Rd, Glasgow, G4 0BA UK; 5 University of Calgary, 2500 University Drive NW, Calgary, AB T2N 1N4 Canada; 6 University of Victoria, 3800 Finnerty Rd, Victoria, BC V8P 5C2 Canada

**Keywords:** Randomized controlled trial, Mobility limitations, Physical activity, Health coaching

## Abstract

**Background:**

Limited mobility in older adults consistently predicts both morbidity and mortality. As individuals age, the rates of mobility disability increase from 1.0% in people aged 15–24 to 20.6% in adults over 65 years of age. Physical activity can effectively improve mobility in older adults, yet many older adults do not engage in sufficient physical activity. Evidence shows that increasing physical activity by 50 min of moderate intensity physical activity in sedentary older adults with mobility limitations can improve mobility and reduce the incidence of mobility disability. To maximize the healthy life span of older adults, it is necessary to find effective and efficient interventions that can be delivered widely to prevent mobility limitations, increase physical activity participation, and improve quality of life in older adults. We propose a randomized controlled trial to assess the effect of a physical activity health coaching intervention on mobility in older adults with mobility limitations.

**Methods:**

This randomized controlled trial among 290 (145 per group) community-dwelling older adults with mobility limitations, aged 70–89 years old, will compare the effect of a physical activity health coaching intervention versus a general healthy aging education program on mobility, as assessed with the Short Physical Performance Battery. The physical activity health coaching intervention will be delivered by exercise individuals who are trained in Brief Action Planning. The coaches will use evidence-based behavior change techniques including goal-setting, action planning, self-monitoring, and feedback to improve participation in physical activity by a known dose of 50 min per week. There will be a total of 9 health coaching or education sessions delivered over 26 weeks with a subsequent 26-week follow-up period, wherein both groups will receive the same duration and frequency of study visits and activities.

**Discussion:**

The consequences of limited mobility pose a significant burden on the quality of life of older adults. Our trial is novel in that it investigates implementing a dose of physical activity that is known to improve mobility in older adults utilizing a health coaching intervention.

**Trial registration:**

ClinicalTrials.gov Protocol Registration System: NCT05978336; registered on 28 July 2023.

## Administrative information

Note: the numbers in curly brackets in this protocol refer to SPIRIT checklist item numbers. The order of the items has been modified to group similar items (see http://www.equator-network.org/reporting-guidelines/spirit-2013-statement-defining-standard-protocol-items-for-clinical-trials/).

**Table:** 

Title {1}	Supporting Physical Activity for Mobility in Older Adults with Mobility Limitations (SuPA Mobility): Study Protocol for a Randomized Controlled Trial
Trial registration {2a and 2b}.	ClinicalTrials.gov Protocol Registration System: NCT05978336.
Protocol version {3}	4
Funding {4}	This study is funded by the Canadian Institutes of Health Research
Author details {5a}	Jordyn Rice^1-3^, Linda C. Li^1,3,4^, Jennifer C. Davis^3,5^, Marco Pahor^6,^ Kenneth Madden^3,7^, Nathan Wei^1-3^, Hubert Wong^8^, Dawn A. Skelton^9^, Sioban McCormick^1-3^, Ryan S. Falck^1-3, 10^, Cindy K. Barha^1-3, 11^, Ryan E. Rhodes^12^, Sohail Loomba^3^, Mohsen Sadatsafavi^13^, Teresa Liu-Ambrose^1-3^ 1. Department of Physical Therapy, Faculty of Medicine, University of British Columbia, Vancouver, British Columbia, Canada. 2. Djavad Mowafaghian Centre for Brain Health, Vancouver Coastal Health Research Institute, Vancouver, British Columbia, Canada. 3. Centre for Aging SMART at Vancouver Coastal Health, Vancouver Coastal Health Research Institute, Vancouver, British Columbia, Canada. 4. Arthritis Research Canada, Vancouver, British Columbia, Canada 5. Applied Health Economics Laboratory, Faculty of Management, University of British Columbia-Okanagan, Kelowna, Canada 6. Institute on Aging, University of Florida, Gainesville, Florida, USA 7. Department of Medicine, Faculty of Medicine, University of British Columbia, Vancouver, British Columbia, Canada 8. School of Population & Public Health, Faculty of Medicine, University of British Columbia, Vancouver, British Columbia, Canada 9. School of Health and Life Sciences, Research Centre for Health (ReaCH), Glasgow Caledonian University, Glasgow, Scotland, UK 10. School of Biomedical Engineering, Faculty of Medicine, University of British Columbia, Vancouver, British Columbia, Canada. 11. Faculty of Kinesiology, University of Calgary, Calgary, AB, Canada 12. School of Exercise Science, Physical and Health Education, University of Victoria, Victoria, British Columbia, Canada 13. Respiratory Evaluation Sciences Program, Faculty of Pharmaceutical Sciences, University of British Columbia, Vancouver, British Columbia, Canada
Name and contact information for the trial sponsor {5b}	Investigator initiated clinical trial: Teresa Liu-Ambrose (Principal Investigator) Teresa.ambrose@ubc.ca
Role of sponsor {5c}	The study sponsor is responsible for overseeing the design, collection, management, data analysis and interpretation, associated publications, and will have ultimate authority over study-related activities.

## Introduction

### Background and rationale {6a}

Decreased physical mobility, like slower walking or taking longer to rise from and sit down in a chair, consistently predict both morbidity and mortality in older adults [1, 2]. Limitations in mobility increase as individuals age, with greater than 20% of individuals over the age of 65 having mobility disability, conceptualized as difficulty getting around one’s environment [3]. Mobility disability rates are higher across all age groups for females, with the largest gap among those aged ≥ 65 years with 22.5% of females reporting mobility disability compared to 18.3% of males [3]. As the aging population continues to rise globally, effective and efficient strategies to combat mobility limitations and disability are critical to reduce burden at the individual, health care system, and societal level.

Physical activity (PA) can improve mobility in older adults with mobility limitations [4]. Limited mobility can be defined as a score of ≤ 9/12 on the Short Physical Performance Battery (SPPB) and is a key risk factor for major mobility disability (MMD), operationalized as the inability to walk 400-m in ≤ 15 min [5]. In a previous randomized controlled trial (RCT), the *Lifestyle Intervention and Independence for Elders* (LIFE), a structured and supervised exercise program significantly reduced the incidence of MMD in sedentary older adults aged 70–89 with mobility limitations [4]. The LIFE study team demonstrated that a minimum effective dose of > 6 min per day (or > 43 min per week) of moderate intensity PA resulted in clinically meaningful effects on mobility and MMD risk in older adults with mobility limitations [6].

Structured and supervised exercise programs, such as the LIFE study intervention [4], benefit mobility and reduce MMD risk but require significant resources that limit widespread deployment. Given the significant impact of limited mobility and MMD [7], we need strategies beyond supervised exercise programs that can be implemented on a broad scale to effectively promote PA participation to improve mobility and decrease the incidence of MMD.

Many older adults with mobility limitations are inactive [8, 9] and do not have the knowledge and skills to increase PA [10]. Health coaching (HC) is “*a patient-centered process that is based upon behavior change theory and is delivered by health professionals with diverse backgrounds*” [11]. Physical activity health coaching uses strategies to promote individual goal-setting and action planning to achieve individualized PA goals and is perceived to be more cost effective than traditional approaches to exercise delivery given it’s potential as a scalable intervention [11]. Physical activity interventions that utilize self-monitoring with feedback have been shown to have the largest effect on PA behaviors (Hedge’s *g* = 0.45, 95% CI: 0.28, 0.63) compared with those that did not (*g* = 0.21, 95% CI: 0.05, 0.35) [12]. A systematic review of 27 RCTs in adults aged ≥ 60 years found HC significantly increased PA participation [13]. However, none of the included RCTs were among older adults with mobility limitations [13], highlighting the void of PA promotion research in this target population at risk for MMD. Thus, we propose that HC will effectively promote PA participation in older adults with mobility limitations and thereby improve mobility and reduce the risk of MMD.

### Objectives {7}

The primary aim of the SuPA Mobility Trial is to assess if a 26-week HC intervention that aims to increase time spent in moderate to vigorous PA (MVPA) by at least 50 min per week can improve mobility, as measured by the SPPB, versus a 26-week health education (ED) program in older adults with mobility limitations. Our secondary objectives are to evaluate the effects of HC compared with ED in the following outcomes: (i) time spent in MVPA and average daily awake sedentary time, (ii) 4-m walk gait speed, (iii) the capacity to complete a 400-m walk in ≤ 15 min (yes/no), (iv) cognitive function, (v) community mobility, (vi) fatigue, (vii) muscle strength, (viii) mood, (ix) quality of life, and (x) sleep. We will also perform a 26-week follow-up assessment to determine whether the benefits of HC versus ED persist 6 months after cessation of the intervention. Additionally, we will perform a concurrent economic evaluation to determine whether HC compared with ED is cost-effective at intervention cessation and follow-up.

### Trial design {8}

**Fig. 1:**
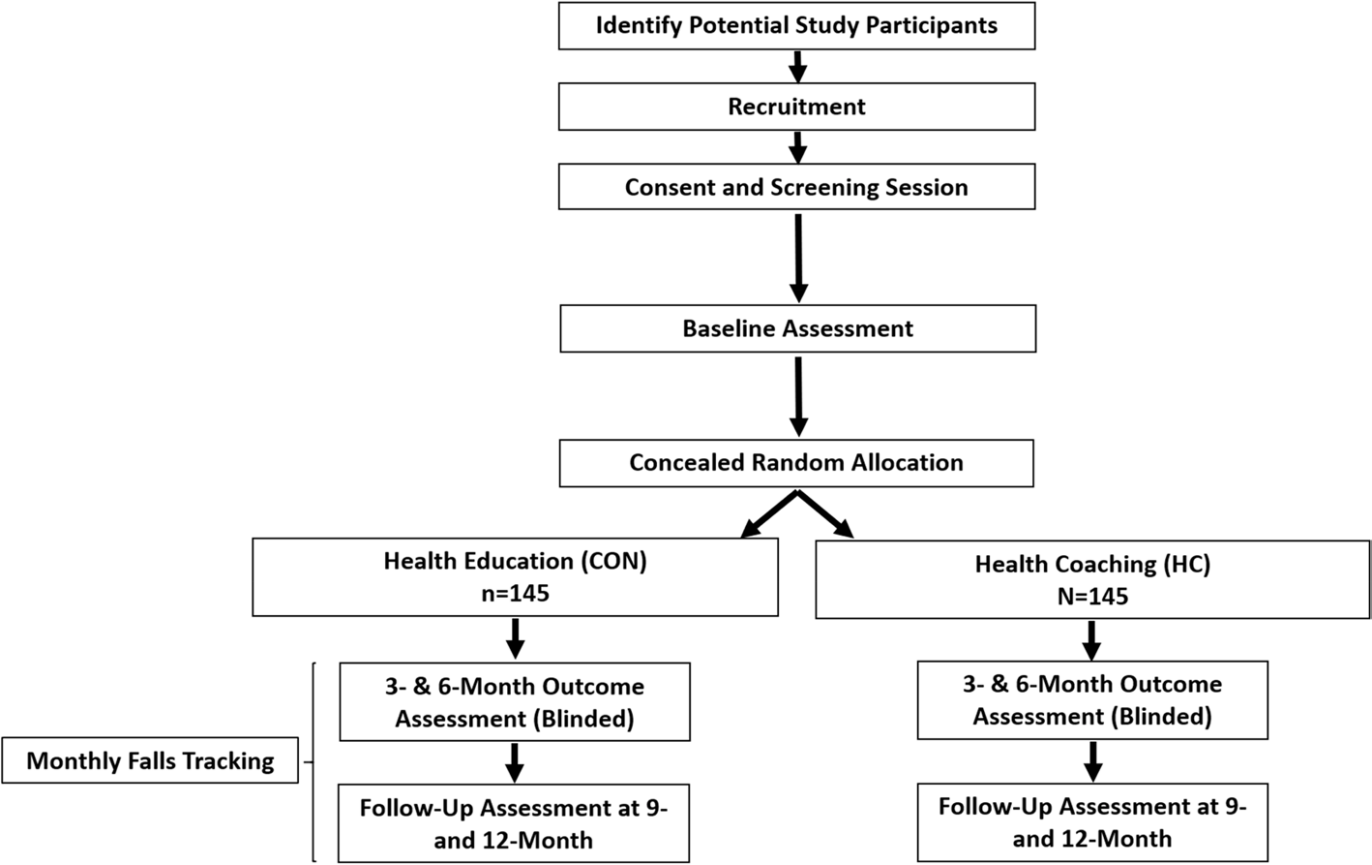
CONSORT study flow of participants

We will conduct a 26-week, parallel group, assessor-blinded, superiority RCT (with 6-month follow-up) in which 290 older adults with mobility limitations will be randomized (1:1) to either (1) health coaching (HC) or (2) health education (ED; attention control). The CONSORT flow of study participants is shown in Fig. 1.

## Methods: participants, interventions and outcomes

### Study setting {9}

The study will be conducted in Vancouver, British Columbia. All study assessments will take place at the Centre for Aging Smart. HC and ED sessions will be performed in person, via zoom, or over the phone. The physical activity intervention will be performed by participants in their desired community setting (e.g., in their own homes) in the greater Vancouver area.

### Eligibility criteria {10}

We will enroll older adults who (1) are aged 70–89 years, (2) score ≤ 9/12 on the SPPB, (3) are able to complete the 400-m walk in ≤ 15 min without sitting and without the help of another person or walker (use of cane is acceptable), (4) score ≥ 22/30 or higher on the Mini-Mental State Examination (MMSE), (5) have no significant functional impairment as indicated by a score of ≥ 6/8 on the Lawton and Brody Instrumental Activities of Daily Living Scale, (6) are able to safely engage in MVPA as determined by the Physical Activity Readiness Questionnaire for Everyone and by the family or study physician if necessary, (7) community-dwelling (i.e., not residing in a nursing home or extended care unit), and (8) able to provide written informed consent. We will exclude individuals who are (1) diagnosed with dementia or stroke; (2) self-report engaging in MVPA ≥ 10 min per week in the prior 3 months; or (3) unable to understand, speak, and read English proficiently.

### Who will take informed consent? {26a}

Trained study personnel will perform the informed consent process with eligible participants. We will obtain written informed consent from study participants prior to engaging in study procedures and ongoing consent will be maintained throughout the trial. Participants will be given the informed consent to review independently prior to meeting in person with staff to go through the consenting procedures.

### Additional consent provisions for collection and use of participant data and biological specimens {26b}

Blood biomarkers will be collected at baseline and 26 weeks in a subset of participants who provide additional consent. Blood will be processed and stored at – 80° C as plasma, serum, and whole blood in a secure research facility.

## Interventions

### Explanation for the choice of comparators {6b}

The ED program will serve as an attention control group to reduce for unknown and known confounding factors such as the effects of attention and social interactions on health outcomes. Social interactions have known gender differences, with women benefitting from social engagement more than men [14]. The ED program will utilize the same participant schedule in frequency and duration of contact as the HC to isolate the specific effects of HC on physical activity.

### Intervention description {11a}

#### Health coaching (HC) intervention

The HC intervention will be delivered over 26 weeks using a Brief Action Planning (BAP) framework to promote PA [15]. Brief Action Planning is grounded in the practice of motivational interviewing and uses evidence-based constructs from the behavior change literature: self-efficacy and action planning [15]. The primary behavior change techniques used are as follows: (1) goal-setting, (2) action planning, (3) self-monitoring, and (4) feedback. Health coaches will facilitate goal-setting and action planning with participants. Participants will utilize daily PA diaries to enable self-monitoring, and the HC will provide feedback on progress toward individualized PA goals and strategies.

The HC intervention will be delivered by exercise professionals (e.g., kinesiologists) with ≥ 1 year of experience working with older adults or clinical populations. All HC will be trained and certified in BAP training by the Centre for Collaboration, Motivation and Innovation [16]. Our HC protocol enables a standardized delivery with an intra-cluster correlation coefficient (ICC) of 0.00071 observed in a previous RCT by a study co-investigator, across 4 BAP trained coaches in adults with knee osteoarthritis [17].

#### Delivery

Participants in the HC intervention will have an initial one-hour session in-person with their coach, who will conduct a brief physical assessment and work with the participant to establish a plan to accomplish their PA goals. Using BAP principles [15], coaches will guide participants to (1) set an activity goal, (2) develop an action plan, (3) identify barriers and solutions, and then (4) rate their confidence in the plan. Coaches will utilize SMART (specific, measurable, attainable, relevant, time-bound) goal setting principles [18, 19]. Once participants have established a goal and plan, they will be asked to rate their confidence in executing the plan on a scale of 0 to 10, with 10 indicating very confident. Collaborative problem solving will occur until the confidence rating reaches ≥ 7/10.

During the initial HC visit, coaches will guide participants to create a plan to increase their participation in MVPA by 50 min per week in 5-, 7-, and 10-min bouts of exercise. Participants will be oriented to various modes of physical activity to increase their MVPA through activities like walking or stationary cycling. The research team will also provide participants with exercise sessions of 5-, 7-, and 10-min durations that will be available as videos and hardcopy manuals. Coaches will teach participants to self-monitor their intensity of PA utilizing the 20-point Borg Rating of Perceived Exertion (RPE). Participants will aim to do moderate to vigorous physical activity with an RPE of 13–14 (“somewhat hard”) on the Borg scale. After the initial HC session, participants will receive 8 × 20-min sessions via zoom or phone calls over the 26-week RCT (Table 1). During these sessions, coaches will continue to help participants set and modify their own exercise goals to gradually increase PA time and intensity and work with participants to create action plans to achieve these goals.

**Table 1:** Overview of HC group

Month	1				2				3				4	5	6
Week	1	2	3	4	5	6	7	8	9	10	11	12	13–16	17–21	22–26
Initial HC session (1 h)	X														
HC phone call (20 min)			X		X		X		X		X		X	X	X
Recommended RPE	11–12				12–13				12–13				13–14		

The fidelity of the HC program will be independently assessed using a multidimensional approach. Audio recordings will be obtained to assess HC delivery and will be rated by an independent coder, not involved in the delivery of the HC intervention, utilizing the Conventry, Aberdeen, & London – Refined (CALO-RE) taxonomy [20]. Informed by Cucciare and colleagues protocol [21], we will analyze recordings from the 1st, 5th (middle), and 10th (last) participant assigned to each coach.

### Health education (ED) program

The ED program will consist of group education sessions delivered in person with an option for people to attend via zoom or phone call. The frequency and duration of ED sessions will be delivered in a manner identical to the HC program, with one initial hour-long session followed by 8 × 20-min education sessions. The initial education topic will be an interactive session on falls prevention. The other education topics will include the following: (1) goal-setting, (2) nutrition, (3) sleep, and (4) mindfulness.

### Criteria for discontinuing or modifying allocated interventions {11b}

Participants will have the goal of increasing PA by 50 min per week, in small doses of 5-, 7-, and 10-min bouts as they choose. In this sense, participants exercise dose will be primarily self-limited based on personal goals and tolerance, with study personnel serving to guide and assist with modification as necessary. Participants who experience falls or report any serious adverse events related or unrelated to the study may require modification of physical activity or require additional medical clearance from the study or their family physician prior to returning to the intervention.

### Strategies to improve adherence to interventions {11c}

HC participants will manually track their weekly PA in a physical log. Health coaches will actively work with participants to help them adjust and modify their action plan to meet their PA goals.

To minimize attrition within the ED group, participants will receive a 1-h HC session and 2 follow-up HC phone calls upon completing the 52-week assessment. At this time, participants will receive access to the exercise intervention videos and manuals.

### Relevant concomitant care permitted or prohibited during the trial {11d}

All participants will seek health care as usual, without any care being prohibited throughout trial. Health care utilization will be assessed via monthly self-report diaries and questionnaires assessed every 3 months throughout the trial duration.

### Provisions for post-trial care {30}

There are no provisions for ancillary and post-trial care. Medical expenses due to care sought for study or non-study-related adverse events will be paid by participant’s usual medical services plan.

### Outcomes {12}

#### Descriptors

We will collect information on key descriptors including general health, biological sex, current medication use, education, and socioeconomic status via interview and questionnaires. Resting vital signs, heart rate and blood pressure, will be measured with an automatic sphygmomanometer, the Omron HEM-775 in a seated position prior to physical assessments. Body mass index will be calculated as mass in kg/height in m [2]. Comorbidity with physical function will be determined by the Function Comorbidity Index [22]. Functional status will be assessed using the Lawton-Brody Instrumental Activities of Daily Living (IADL) Scale [23]. Gender-related characteristics will be assessed using questionnaires about gender identity (Gender Identity Scale) [24] and institutional gender (income, education level). The Gender Identity Scale offers three gender identities: female/woman/girl, male/man/boy, and other gender(s) [24].

All outcomes will be assessed at baseline, 13 weeks (mid-point), 26 weeks (final), and 52 weeks (follow-up). The following secondary outcomes will also be collected midway through the follow-up period at 39 weeks via phone call: fatigue, community mobility, mood, health-related quality of life, and sleep.

### Primary outcome

#### Mobility

The primary outcome is mobility, measured by the SPPB. The SPPB is a standardized measure of lower extremity physical performance that includes standing balance, walking, and sit-to-stand [25]. Each component is rated out of 4 points, for a maximum score of 12 points, with higher scores indicating better performance. A low score of 9 or less on the SPPB is a risk factor for institutionalization, morbidity, mortality, and disability in non-disabled older adults [26]. Specifically, scores of 9 or less on the SPPB are predictive of MMD [27].

### Secondary outcomes

#### Physical activity

Average daily MVPA and sedentary time will be measured using the SenseWear Mini, a research-grade multimodal sensor. The SenseWear Mini integrates tri-axial accelerometer data, physiological sensor data, and personal demographic information to provide valid and reliable estimates of steps and energy expenditure in metabolic equivalent of task (MET) [28, 29]. We will calculate MVPA as the average daily minutes spent with an energy expenditure of ≥ 3 MET, the lower bound of MVPA. Sedentary time will be defined as the average daily minutes spent with an energy expenditure of ≤ 1.5 MET during waking hours. Participants will wear the device around their non-dominant upper arm for 9 days at baseline and 3 and 6 months. Data from the last 7 days of wear will be utilized for analysis to minimize potential effects of the device temporarily increasing their PA levels [30]. An alternative tri-axial accelerometer-based wearable may be used for those who experience contact dermatitis with the SenseWear Mini.

The Community Health Activities Model Program for Seniors (CHAMPS) Physical Activity Questionnaire for Older Adults will be used as a paper and pencil measure of physical activity. The CHAMPS is a 41-item questionnaire assessing weekly frequency and duration of physical activities relevant for older adults [31].

#### Gait speed

Gait speed in m/s will be assessed during two trials of the 4-m walk performed during the SPPB assessment.

#### 400-m walk

Participant’s capacity to perform the 400-m walk overtime will be assessed by whether participants can complete the 400-m in ≤ 15 min and will be recorded as “yes” or “no.” Capacity to complete the 400-m walk is a major risk factor for major mobility disability. During the 400-m walk, participants will be asked to walk 10 laps (out and back) on a 20-m course at their usual pace [32]. Participants will be able to use a cane, but no further physical assistance or walking aid will be permitted. If needed, they may stop for a standing rest of up to 1 min during the test. During the 400-m walk participants heart rate will be continuously measured using a wearable device.

#### Cognitive function

We will use a battery of standardized neuropsychological tests to assess multiple cognitive domains. The National Institutes of Health (NIH) Toolbox Cognition battery [33, 34] is a comprehensive computerized neuropsychological test battery with normative values. We will use the following assessments from the NIH Toolbox: (1) Dimensional Card Sorting to measure set shifting, (2) Flanker Inhibitory Control and Attention Test to measure response inhibition and attention, (3) List Sorting Working Memory Test to measure working memory, and (4) Picture Sequence Memory Test to measure episodic memory. Additionally, we will use the following paper and pen neuropsychological assessments: (1) 13-item Alzheimer's Disease Assessment Scale-Cogniive (ADAS-Cog), [35, 36] (2) Trail Making Test Parts A and B (B-A; set shifting), [37] (3) Digit Span Forward and Backward (working memory), [38] (4) Rey Auditory Verbal Learning Test (verbal memory), [37] (5) the Digit Symbol Substitution Test (processing speed), [39] (6) Stroop Colour Word Test (response inhibition), [40] (7) Category Fluency, (8) Clock Drawing, and (9) MoCA [41]. The paper and pen neuropsychological battery will allow us to calculate the ADAS-Cog-Plus [35].

#### Fatigue

The 9-item Fatigue Severity Scale [42] will be used to assess how fatigue interferes with certain activities and its severity.

#### Strength

Dominant quadriceps strength in kilograms will be measured with a standardized strain gauge. Dominant grip strength in Newtons will be measured using a digital Jamar isometric hand dynamometer. Participants will perform three trials each for quadriceps and grip strength assessments.

#### Functional and community mobility

Participants will perform two trials of the Timed Up-and-Go Test to assess functional mobility [43]. Community mobility will be assessed using the Life Space Questionnaire [44].

#### Mood

We will use the Centre for Epidemiological Studies Depression Scale, a commonly used screening tool for depressive symptoms [45].

#### Health-related quality of life

A self-report questionnaire, the EuroQol EQ-5 Domain (5D)-5 Level (5L) (EQ-5D-5L), will be utilized to assess health-related quality of life [46–48]. The EQ-5D-5L is a preference-based utility measure incorporating five domains of health: mobility, self-care, usual activity, pain, and anxiety/depression. Higher scores indicate a greater severity of problems within the domain. We have used the EQ-5D previously for a cost-utility analyses in older adults with mobility limitations [9, 49]. It calculates health state utility values that can provide weightings for quality-adjusted life years (QALYs). Canadian conversion tariffs will be used to estimate health state utility values [47].

#### Sleep

Objective sleep duration and sleep efficiency will be measured using the SenseWear Mini [50]. Sleep quality will be assessed using the 19-item Pittsburgh Sleep Quality Index [51], a commonly used subjective sleep questionnaire. Participants will also keep a self-report sleep diary which will be used to confirm sleep windows calculated from the SenseWear Mini. The STOP-Bang questionnaire will be used to screen for risk of obstructive sleep apnea at baseline [52].

#### Falls

Falls will be prospectively documented by all participants on a monthly basis via calendars provided by the research team. Participants will be asked to report any falls directly to the research team after they occur. We will complete a follow-up falls interview over the phone to obtain information regarding the fall and determine if any adjustments to the study protocol and interventions are required.

#### Health resource utilization (HRU)

Cost data will be collected monthly via HRU diaries, and these self-report cost diaries will be confirmed during in person assessments or via telephone calls every 3 months using the HRU questionnaire [53], which is based on validated cost questionnaires [54–56]. The HRU questionnaire is utilized to collect specific details on (1) health professional visits; (2) hospital, rehabilitation facility, and inpatient clinic visits; (3) laboratory procedures or investigations; and (4) medications. Using a fully allocated hospital cost model (for in-patient costs) and the British Columbia provincial guide to medical fees (for outpatient costs), we will assign health care resource utilization on a per participant basis. Evidence from a systematic review of 15 studies demonstrates good agreement between self-reported questionnaires on resources utilization and administrative data [57].

#### Intervention adherence

Adherence to the intervention will be measured as session attendance (HC and ED groups). Session attendance will be recorded by health coaches and education session leaders and will be estimated as the percentage of total sessions attended.

### Participant timeline {13}

Participant enrolment began July 2023 and the first participant was randomized September 5, 2023. We expect participant recruitment, assessments, and follow-up visits to be completed by October 2026. Participants will undergo a phone screening to determine preliminary eligibility. If appropriate, participants will be invited for an in-person screening. Within 1 month of the in-person screening, participants will complete the remainder of the baseline assessments and will be randomized to either the HC or ED group. Participants will partake in intervention procedures for 26 weeks. Upon completing the intervention, participants will be followed for another 26 weeks. Upon the final follow-up assessment at 52 weeks, participants in the ED group will receive an initial HC visit followed by 2 additional HC calls and receive access to exercise-related materials. The participant timeline of assessments is shown in Table 2.

**Table 2:**
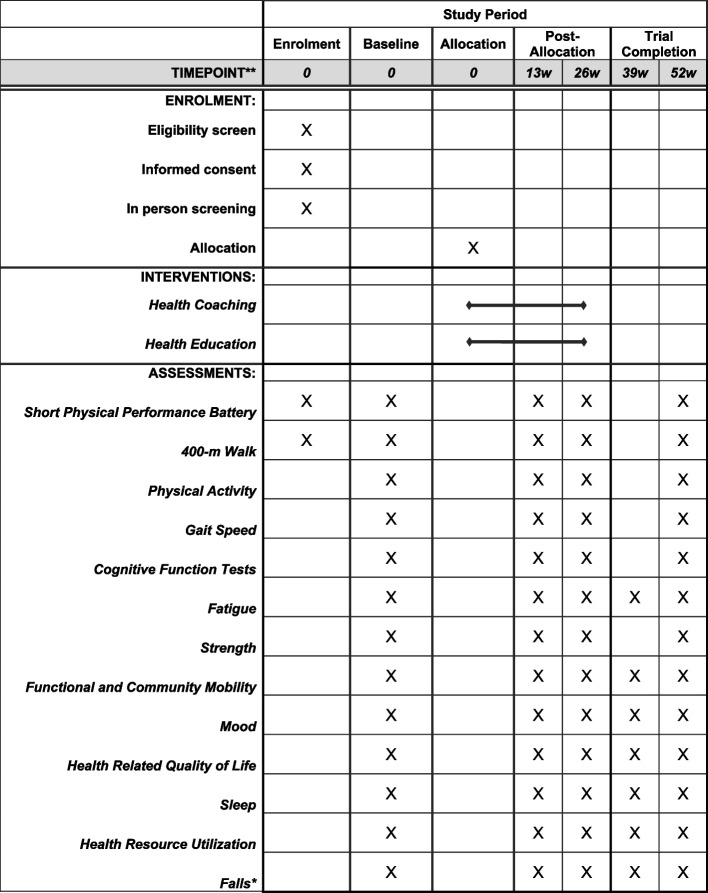
Participant timeline of assessments

*Falls data will be collected monthly throughout the trial

### Sample size {14}

The sample size for this study is 290 participants, based on SPPB data published in the LIFE study that examined changes in SPPB scores based on changes in MVPA [6]. At 6 months, those in the highest quartile of MVPA change (i.e., > 43 min per week) experienced a mean improvement (calculated as 6 months minus baseline) of 1.21 in SPPB score with a SD = 0.98. A 0.5 difference in SPPB score is considered a clinically meaningful change [58]. Assuming at least 90% of our HC participants will increase their participation in MVPA by at least 50 min per week, and splitting the remaining 10% of HC participants equally among the 3rd quartile in MVPA change (mean SPPB change = 1.16) and the 2nd quartile in MVPA change (mean SPPB change = 0.89), we anticipate a mean SPPB improvement of 0.90 × 1.21 + 0.05 × 1.16 + 0.05 × 0.89 = 1.19 in the HC group. For the ED group, we assume the mean SPPB change will be similar to what was observed in the LIFE study control group [6]; we calculated this value to be 0.84 from the published data. Thus, the effect size for the SPPB outcome is (1.19 − 0.84)/0.98 = 0.36.

Using a two-tailed test alpha = 0.05, our estimated effect size requires a sample size of 246 participants (123 per group) in order to achieve 80% power, assuming the outcomes for all participants are independent. In principle, this sample size should be adjusted by a design effect (DE) to account for the nesting of participants within coaches in the HC arm and nesting of participants within cohorts in the ED arm. However, as noted in the description of the HC intervention, the ICC in a previous study was found to be only 0.0007 [17]. Together with an expected 15 participants per coach, the DE is only 1 + (15 − 1) × 0.007 = 1.01 which implies a negligible impact on the required sample size. No data are available on the ICC within ED groups, but we expect it to be low enough to also have negligible impact. Allowing for a 15% dropout rate leads to the sample size of 290 participants (i.e., 145/group).

### Recruitment {15}

We will recruit participants from 2 committed clinics: (1) Vancouver General Hospital (VGH) Falls Prevention Clinic and (2) VGH Geriatric Internal Medicine Teaching Clinic. Additionally, we will recruit participants through word of mouth, public talks, and advertisements place in community centers and newspapers. We have an established track record of recruiting older adults, including those with mobility limitations, for behavioral RCTs.

## Assignment of interventions: allocation

### Sequence generation {16a}

Participants will be allocated to experimental groups (1:1 ratio) in blocks formed by sequentially enrolled participants. Block sizes may vary to accommodate varying recruitment rates over time and ensure participants receive timely intervention. Participants within each block are randomized into ED to form a cohort for the group education sessions. The allocations will be generated in 'R' using a fixed seed to ensure reproducibility of the random sequence.

### Concealment mechanism {16b}

When a block has been filled, the study coordinator will send the participant study identifiers (ID) to the study statistician, who is not involved in the RCT. The statistician will attach the allocations and return the list to the study coordinator.

### Implementation {16c}

Upon completion of the baseline assessments, the study coordinator will reveal which experimental group participants are been assigned to for scheduling purposes.

## Assignment of interventions: blinding

### Who will be blinded {17a}

Assessments will be performed by blinded study personnel who are not involved in the HC or ED interventions or study enrolment. Blinding of participants will not be feasible. Participants will be reminded to not discuss or disclose their study activities with assessors.

### Procedure for unblinding if needed {17b}

N/A. There is no indication of unblinding for the assessors.

## Data collection and management

### Plans for assessment and collection of outcomes {18a}

Research personnel will be trained in all primary and secondary outcome measure collection to minimize intra- and inter-rater variations. Outcomes will be collected at 4 time points throughout the trial: (1) baseline (at enrolment), (2) midpoint (13 weeks), (3) final (26 weeks), and (4) follow-up (52 weeks).

### Plans to promote participant retention and complete follow-up {18b}

Participants in the HC group will have regular communication from bi-monthly to monthly sessions with their BAP coach to promote retention and adherence to the intervention. Participants in the ED group will have the same structure of engagement with group education sessions to promote retention and adherence. Education session attendance will be incentivized by entering participants into a gift card drawing every other session. Throughout the study intervention and follow-up time frame, participants will receive monthly communication from study personnel to assess falls and other outcomes. In a previous study utilizing a similar HC protocol in adults with mild cognitive impairment, adherence rates to PA recommendations with ≥ 73% and attrition rates were ≤ 10.4% [59, 60].

### Data management {19}

Data will be managed using Research Electronic Data Capture (REDCap) which is a secure web-based application. Data will be entered by trained study personnel who will conduct range checks for data values to ensure quality.

### Confidentiality {27}

Only the PI and approved study personnel will have access to the data and no unauthorized personnel will be able to access the data. Physical copies of data will be stored in a secured and locked location, and electronic documents will be stored on a secured server. All participant data will be associated with an identification code and not by personal name or details. There will be one document that maps participant names to their identifier, which will be stored securely on the server and password protected so that only the PI and research coordinator will have access to this information.

### Plans for collection, laboratory evaluation and storage of biological specimens for genetic or molecular analysis in this trial/future use {33}

Research blood will be collected in a subset of participants, based on funding. The analytes of interest will include neurotrophic factors, myokines, sex steroid hormones, and pro- and anti-inflammatory cytokines. Whole blood, serum, and plasma will be processed and stored at − 80 °C in a secure research facility. Remaining blood samples will be stored for 5 years from collection and then destroyed.

## Statistical methods

### Statistical methods for primary and secondary outcomes {20a}

All primary and secondary analysis will follow the intent to treat principle. Participants who have been randomized will be included in all analysis to estimate intervention effects irrespective of deviations from the intervention protocol [61]. The primary outcome (SPPB) will be modeled using a three-level linear mixed model that includes random intercepts at the coach and the participant levels (for the HC group) or the education cohort and the participant levels (for the ED group), fixed effects of time, experimental group (HC vs. ED), and the group-by-time interactions. The treatment effect will be estimated as the difference in mean change in outcome between the two arms, calculated using the fitted model coefficients. Secondary outcomes will be analyzed using analogous linear mixed models. All linear mixed models will be adjusted for baseline outcome score and baseline characteristics. The proportion of participants able to complete the 400-m walk at trial completion and at 6-month follow-up will be compared using a two-level (clusterd by coach in the HC group, clusterd by cohort in the ED group) logistic mixed model for binary outcomes. A pre-specified statistical analysis plan will be developed prior to the release of data for analysis.

### Interim analyses {21b}

N/A. No other interim analyses are planned.

### Methods for additional analyses (e.g., subgroup analyses) {20b}

We will conduct a cost-effectiveness analysis and a cost-utility analysis with the incremental cost-effectiveness ratio (ICER) and the incremental cost-utility (ICUR) as the primary economic outcomes [62]. The ICER represents the difference between the mean cost of the HC intervention compared with the ED program, divided by the difference in mean effectiveness (measured by the SPPB), where the ICER = ∆ Cost/∆ SPPB (where a change of 0.5 in the SPPB score is clinically meaningful). The ICER represents the difference between the mean cost of the HC intervention compared with the ED, divided by the difference in mean utility (measured by QALYs), where the ICUR = ∆ Cost/∆ QALY. QALYs will be estimated using the EQ-5D-5L and area under the curve analysis. We will determine the incremental cost per mean change in QALYs estimated from the EQ-5D-5L of HC versus ED. This economic evaluation will use a 26-week time horizon that aligned with intervention cessation and 52 weeks that aligns with the 26-week follow-up. The economic evaluation will be completed using two perspectives, the health care system and societal [62].

We will perform a subgroup analysis including the interaction term of biological sex by experimental group (sex x group) to account for possible effects of sex on the outcomes of the intervention. Outcomes will also be analyzed by gender by including the interaction term of gender identity, using the Gender Identity Scale [24], by experimental group (gender x group).

### Methods in analysis to handle protocol non-adherence and any statistical methods to handle missing data {20c}

Missing outcome data will not be imputed since estimates from linear mixed models are fully efficient if the missing data are missing at random [63]. If we encounter more than 10% missing outcome data, we will consider plausible explanations for why the data may not be missing at random and assess the sensitivity of the results by fitting a joint model that incorporates non-ignorable dropout [64]. Missing covariate data will be multiply imputed assuming they are missing at random, unless there is a clear rationale for treating them otherwise.

### Plans to give access to the full protocol, participant level-data and statistical code {31c}

Upon completion of study procedures and the primary study publications, the full protocol will be uploaded to ClinicalTrials.gov. Data sharing with the broader scientific community will be prioritized as determined by the PI, based on reasonable requests. All data that is published or shared will remain deidentified and participant privacy and confidentiality will be maintained.

## Oversight and monitoring

### Composition of the coordinating center and trial steering committee {5d}

A joint trial steering and data safety monitoring committee (TSC and DSMC) will be made up of an independent chair and two external members not involved in day-to-day conduct of the research trial. The TSC will monitor RCT progress, identify challenges, and facilitate mitigation strategies with the PI.

### Composition of the data monitoring committee, its role and reporting structure {21a}

The DSMC will review adverse events reported by participants, stop the study trial if warranted by data, and advise PI on measures needed to ensure participant safety throughout the trial.

### Adverse event reporting and harms {22}

Adverse events will be collected during monthly phone calls to participants during the 26-week intervention. In case of a serious adverse event during the study, participants will be instructed to call 911 or report to the nearest emergency room. The TSC/DSMC will review all adverse events every 3 months and classify them based on the definitions from the January 2007 OHRP *Guidance on Reviewing and Reporting Unanticipated Problems involving Risks to Subject or Others and Adverse Events, OHRP Guidance*. The TSC/DSMC will stop the study or advise on modifications to the protocol to maintain safety as warranted by the data.

### Frequency and plans for auditing trial conduct {23}

The joint TSC and DSMC will meet every 3 months to review safety data and monitor RCT progress. As this is a minimal risk study, the University of British Columbia Research Ethics Board will review trial conduct on an annual basis.

### Plans for communicating important protocol amendments to relevant parties (e.g., trial participants, ethical committees) {25}

Any protocol amendments recommended by the TSC/DSMC and approved by the research ethics board that impact study participants will be discussed with individuals in the trial as part of maintaining continual active informed consent. Approved protocol amendments will be updated on ClinicalTrials.gov.

### Dissemination plans {31a}

The results of this study will be utilized to make evidence-based recommendations to promote PA in older adults to promote mobility and prevent disability. We plan to disseminate our results widely through targeting our dissemination efforts to 3 primary groups: (1) older adults and community stakeholders, (2) clinicians and health practitioners, (3) and researchers.

Through our established relationships within the community, we will host public talks to share our findings with stakeholders. We plan to organize a 2-day scientific symposium in collaboration with the Centre for Aging Smart and Vancouver Coastal Health Research Institute to exchange updates on mobility in aging with leaders in the scientific community. A companion forum will be held in a similar fashion to disseminate results to the lay public and broader community health professionals. Study results will be shared broadly through conference presentations and peer-reviewed publications as well as through an established network, Physical Activity for Precision Health [65], a network of Canadian and international researchers, which includes a Patient Advisory Group.

## Discussion

Previous research has demonstrated the importance of MVPA on improving mobility and reducing MMD risk in older adults with mobility limitations. However, there is a need to identify strategies that can be delivered at scale for this population. The proposed research will utilize a novel HC intervention to promote MVPA by a minimum effective dose in older adults with mobility limitations. The findings of this study will provide insights on the effect of a HC intervention on mobility in older adults with mobility limitations and thus, at risk for MMD. This study will evaluate effects of HC on secondary health outcomes, including PA levels, gait speed, 400-m walk, cognitive function, fatigue, strength, functional and community mobility, mood, quality of life, sleep, and falls. Finally, this study will provide an estimate of the cost-effectiveness of the HC intervention. These findings have the potential to reduce the personal and societal burden of limited mobility and MMD via a strategy that can be widely implemented.

### Trial status

This protocol was originally approved May 1, 2023 (Version 3). The first participant was randomized September 5, 2023, and study-related visits are expected to be completed by October 2026.

## Data Availability

The final data set will remain within the study team under the primary responsibility of the primary investigator, who will determine sharing of the data set upon reasonable request.

## Abbreviations

ED: Health education program
HC: Health coaching
MMD: Major mobility disability
MVPA: Moderate to vigorous physical activity
PA: Physical activity
SPPB: Short Physical Performance Battery
